# Neutrophil Percentage-to-Albumin Ratio: A Good Parameter for the Evaluation of the Severity of Anti-NMDAR Encephalitis at Admission and Prediction of Short-Term Prognosis

**DOI:** 10.3389/fimmu.2022.847200

**Published:** 2022-04-11

**Authors:** Yao Tang, Haiman Hou, Lanjun Li, Liuliang Yong, Shuang Zhang, Lulu Yan, Xiaoxue Huang, Jun Wu

**Affiliations:** ^1^ Department of Neurology, The First Affiliated Hospital of Zhengzhou University, Zhengzhou, China; ^2^ Department of Radiology, The First Affiliated Hospital of Zhengzhou University, Zhengzhou, China

**Keywords:** anti-NMDAR encephalitis, neutrophil percentage-to-albumin ratio, anti-NMDAR antibody, disease severity, neutrophil, albumin, prognosis

## Abstract

**Objectives:**

The purpose of this study was to investigate the association of neutrophil percentage-to-albumin ratio (NPAR) with the severity at admission and discharge (short-term prognosis) in patients with anti-N-methyl-D-aspartic acid receptor (NMDAR) encephalitis.

**Methods:**

Multivariable logistic regression models such as NPAR were constructed based on univariable regression results. Receiver operating characteristic (ROC) curves, nomograms, and concordance index (c-index) were used to evaluate the efficacy of the models in assessing disease severity at admission and predicting short-term prognosis, validated by bootstrap, Hosmer–Lemeshow goodness-of-fit test, calibration curves, and decision curve analysis.

**Results:**

A total of 181 patients with anti-NMDAR encephalitis diagnosed at the First Affiliated Hospital of Zhengzhou University were included. The results showed that NPAR had good sensitivity and specificity in assessing disease severity at admission and predicting short-term prognosis. The multivariable logistic regression models based on NPAR and other influencing factors had good discrimination, consistency, accuracy, calibration ability, applicability, and validity in assessing the severity at admission and predicting short-term prognosis.

**Conclusion:**

NPAR has good clinical value in assessing disease severity at admission and predicting short-term prognosis of patients with anti-NMDAR encephalitis.

## Introduction

Autoimmune encephalitis is a kind of encephalitis mediated by autoimmune mechanisms. Anti-N-methyl-D-aspartate receptor (NMDAR) encephalitis is the most common type of autoimmune encephalitis and is potentially lethal. It is characterized by psychiatric behavior, seizures, movement disorder, autonomic dysfunction, and central hypoventilation. Anti-NMDAR antibodies are essential for the diagnosis of anti-NMDAR encephalitis, but its value in the assessment of the severity and prognosis is still controversial (1). Besides, there are cases with false positive and false negative results. Currently, there is a lack of biological indicators that can adequately evaluate the severity and prognosis of anti-NMDAR encephalitis.

Neutrophils are often altered in inflammatory and immune diseases, and lymphocytes are important immune cells of the body. They make up a major proportion of the leukocyte population, and the percentage of neutrophils and lymphocytes often fluctuates from one to the other. Therefore, neutrophil percentage not only directly reflects the ratio of neutrophils but also indicates the ratio of lymphocytes to a certain extent. Several studies have shown that albumin is also associated with the severity and prognosis of autoimmune encephalitis (2). In recent years, an association has been found between neutrophil percentage-to-albumin ratio (NPAR) and septic shock (3), acute kidney injury (4), bladder cancer (5), cardiogenic shock (6), and stroke-associated infection (7). However, its role in anti-NMDAR encephalitis has not been reported.

This study intended to summarize and analyze the NPAR level in patients with anti-NMDAR encephalitis, and to investigate its value in assessing disease severity at admission and at discharge (short-term outcome).

## Methods

### Research Subjects

Patients with anti-NMDAR encephalitis diagnosed in the First Affiliated Hospital of Zhengzhou University from January 2014 to June 2021 were enrolled in the study. This retrospective study was reviewed, approved by the Ethics Committee of the First Affiliated Hospital of Zhengzhou University (Number: 2021-KY-0974-002).

### Inclusion Criteria and Exclusion Criteria

**Figure 1:**
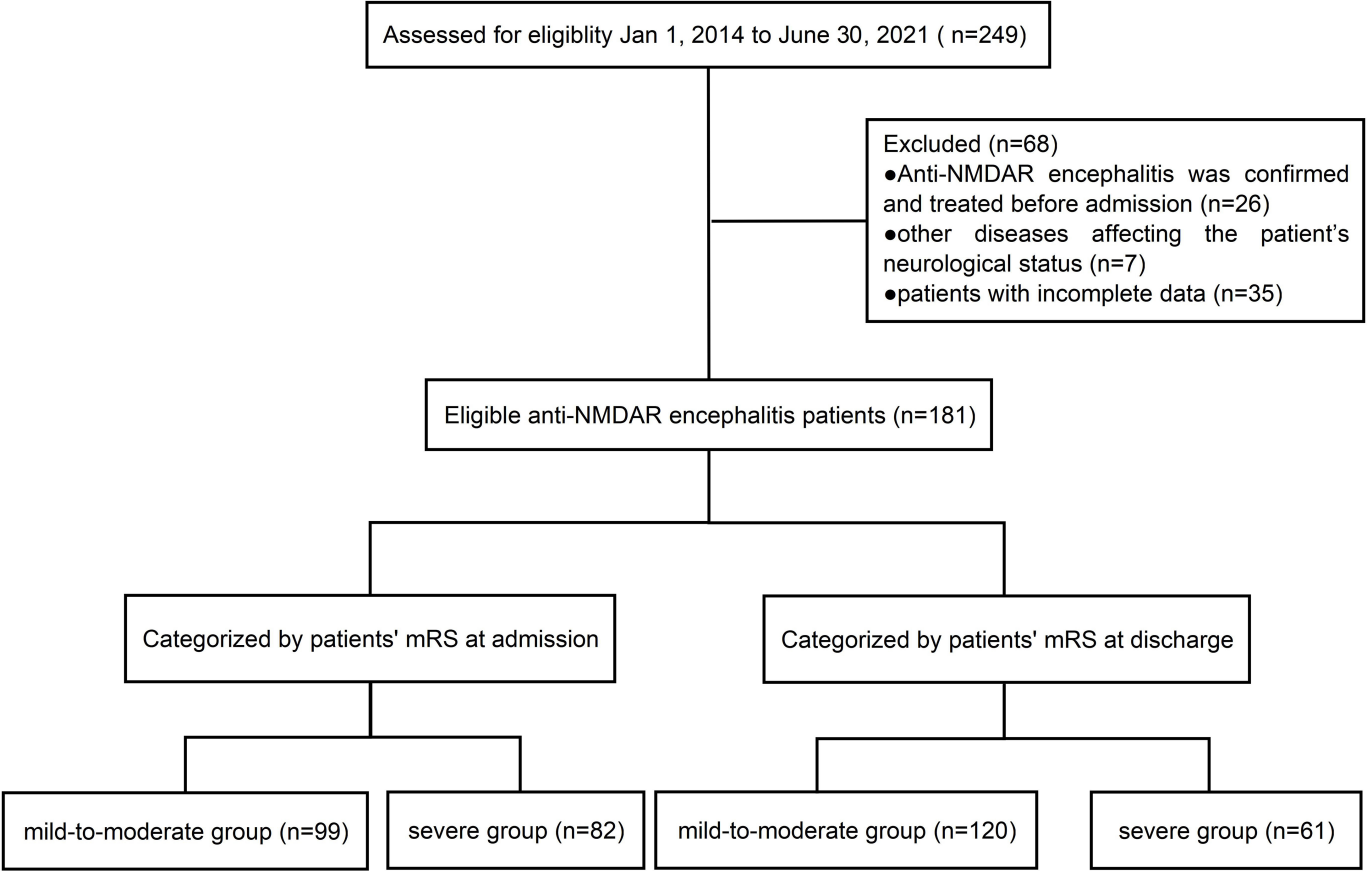
Cohort selection process of the study population.

The inclusion criteria were as follows: (1) patients had at least one of the following symptoms: abnormal psychiatric behavior/cognitive impairment, seizures, speech dysfunction, involuntary movement, consciousness declination, autonomic dysfunction/central hypoventilation; (2) anti-NMDAR antibodies were tested positive in cerebrospinal fluid (CSF); and (3) other diseases were reasonably excluded. The exclusion criteria included the following: (1) anti-NMDAR encephalitis was confirmed and treated in other healthcare centers before admission (26 patients); (2) other diseases affecting the patients’ neurological status (7 patients); and (3) patients with incomplete data (35 patients) (**Figure 1**).

### Research Methods

Demographic data included gender and age. Clinical features and therapy included time from onset to admission, anti-infective therapy before admission, prodromal symptoms, main clinical manifestations [data collected from onset to admission and onset to discharge respectively, including seizures, psychiatric behavior, involuntary movement, autonomic dysfunction, consciousness declination, cognitive dysfunction, speech dysfunction, admission to intensive care unit (ICU)], tumor presence, pneumonia, immunotherapy, and duration of hospital stay. Auxiliary examination included count of white blood cell (WBC), C-reactive protein (CRP), neutrophil percentage, albumin within 24 h of admission, anti-NMDAR antibodies in serum and CSF, other biochemical and cytological tests in CSF, and brain magnetic resonance imaging (MRI). Then, NPAR was calculated. Neurological function was assessed using the modified Rankin scale (mRS). mRS score ≤3 was classified as mild to moderate, and mRS score >3 was classified as severe.

### Statistical Methods

Normally distributed data were expressed as the mean ± standard deviation (SD), and independent sample t-test was used to compare data. Non-normally distributed data were presented as the median (IQR: interquartile range) and were compared using the Mann–Whitney U test. Categorical data were presented as the number of cases (percentage) and were compared using chi-squared tests. The indexes of a single-factor analysis of p < 0.05 were included in the multivariable logistic regression analysis. Receiver operating characteristic (ROC) curves were calculated to compare the diagnostic value of clinical test parameters and determine their critical value. According to the results of multivariable logistic regression analysis, nomograms were established. Bootstrap was used to internally validate. Concordance index (c-index), ROC curves, calibration curves, Hosmer–Lemeshow goodness-of-fit test, and decision curve analysis were used to assess the predictive value of the models. Results were considered statistically significant at p < 0.05. These statistical analyses were performed using SPSS 21.0, GraphPad Prism 8, MedCalc 19, and R software.

## Results

The clinical data of 181 patients were presented in **Tables 1** and **2**. The study consisted of 90 males (49.7%) and 91 females (50.3%) with a median age of 25 years (IQR 17–37). 82 patients (45.3%) were admitted with mRS >3, and 61 patients (33.7%) were discharged with mRS >3. The median neutrophil percentage was 76.6 (IQR 65.9–83.7). The mean of albumin and NPAR was, respectively, 42.3 (SD 4.6) and 1.8 (SD 0.4).

**Table 1:** Comparison of clinical data of anti-MNDAR encephalitis with different severity at admission.

	Total (n = 181)	mRS ≤3 (n = 99)	mRS >3 (n = 82)	*P* value
**Demographic data**				
Gender				0.085
Male, n (%)	90 (49.7)	55 (55.6)	35 (42.7)	
Female, n (%)	91 (50.3)	44 (44.4)	47 (57.3)	
Age, median (IQR)	25 (17–37)	23 (15–35)	27.5 (19–41.3)	0.06
**Clinical features from onset to admission**				
Time from onset to admission, median (IQR)	15 (8–26)	15 (8–30)	14.5 (7.8–20)	0.219
Anti-infective therapy before admission, n (%)	85 (47)	42 (42.4)	43 (52.4)	0.179
Prodromal symptoms, n (%)	114 (63)	65 (65.7)	49 (59.8)	0.413
Seizures, n (%)	83 (45.9)	40 (40.4)	43 (52.4)	0.106
Psychiatric behavior, n (%)	131 (72.4)	68 (68.7)	63 (76.8)	0.223
Involuntary movement, n (%)	16 (8.8)	7 (7.1)	9 (11)	0.357
Autonomic dysfunction, n (%)	90 (49.7)	38 (38.4)	52 (63.4)	0.001
Consciousness declination, n (%)	75 (41.4)	13 (13.1)	62 (75.6)	<0.001
Cognitive dysfunction, n (%)	63 (34.8)	32 (32.3)	31 (37.8)	0.441
Speech dysfunction, n (%)	29 (16.0)	18 (18.2)	11 (13.4)	0.384
ICU admission, n (%)	73 (40.3)	14 (14.1)	59 (72)	<0.001
Tumor presence, n (%)	15 (8.3)	5 (5.1)	10 (12.2)	0.083
**Auxiliary examination**				
Abnormal MRI, n (%)	68/163 (41.7)	32/91 (35.2)	36/72 (50)	0.056
CSF tests				
Immunoglobulin quotient, n (%)	15/139 (10.8)	8/77 (10.4)	7/62 (11.3)	0.865
Albumin quotient, n (%)	31/139 (22.3)	17/77 (22.1)	14/62 (22.6)	0.944
CSF with oligoclonal bands, n (%)	36/139 (25.9)	16/77 (20.8)	20/62 (32.3)	0.125
CSF WBC, n (%)	97/140 (69.3)	49/76 (64.5)	48/64 (75)	0.179
CSF lymphocyte, n (%)	116/140 (82.9)	63/76 (82.9)	53/64 (82.8)	0.99
CSF protein, n (%)	45/140 (32.1)	27/76 (35.5)	18/64 (28.1)	0.35
Blood tests				
WBC, median (IQR), ×10^9^/L	9 (7-11.7)	8.4 (6.2-10.2)	11 (8.1-13.5)	<0.001
Neutrophil percentage, median (IQR), %	76.6 (65.9–83.7)	70 (60–78.6)	82.7 (76.5–87.6)	<0.001
Albumin, mean (SD), g/L	42.3 (4.6)	44.4 (3.5)	39.8 (4.6)	<0.001
NPAR, mean (SD)	1.8 (0.4)	1.5 (0.3)	2.1 (0.4)	<0.001
CRP, median (IQR), mg/L	2.2 (0.6–7.8)	1.1 (0.4–2.6)	4.8 (1.4–16.7)	<0.001
Serum anti-NMDAR antibody, n (%)	75/116 (64.7)	37/60 (61.7)	38/56 (67.9)	0.486

mRS, modified Rankin scale; IQR, interquartile range; ICU, intense care unit; MRI, magnetic resonance imaging; CSF, cerebrospinal fluid; WBC, count of white blood cell; SD, standard deviation; NPAR, neutrophil percentage-to-albumin ratio; CRP, C-reactive protein; NMDAR, N-methyl-D-aspartate receptor.

**Table 2:** Comparison of clinical data of anti-MNDAR encephalitis with different severity at discharge (short-term prognosis).

	Total (n = 181)	mRS ≤ 3 (n = 120)	mRS >3 (n = 61)	*P* value
**Demographic data**				
Gender				0.094
Male, n (%)	90 (49.7)	65 (54.2)	25 (41)	
Female, n (%)	91 (50.3)	55 (45.8)	36 (59)	
Age, median (IQR)	25 (17–37)	24 (16–34.8)	29 (19–42.5)	0.054
**Auxiliary examination**				
Abnormal MRI, n (%)	68/163 (41.7)	43/110 (39.1)	25/53 (47.2)	0.327
CSF tests				
Immunoglobulin quotient, n (%)	15/139 (10.8)	8/97 (8.2)	7/42 (16.7)	0.142
Albumin quotient, n (%)	31/139 (22.3)	25/97 (25.8)	6/42 (14.3)	0.135
CSF with oligoclonal bands, n (%)	36/139 (25.9)	21/97 (21.6)	15/42 (35.7)	0.082
CSF WBC, n (%)	97/140 (69.3)	58/90 (64.4)	39/50 (78)	0.096
CSF lymphocyte, n (%)	116/140 (82.9)	72/90 (80.0)	44/50 (88)	0.229
CSF protein, n (%)	45/140 (32.1)	32/90 (35.6)	13/50 (26)	0.246
Blood tests				
WBC, median (IQR), ×10^9^/L	9 (7-11.7)	8.4 (6.4-10.6)	11 (9-13)	<0.001
Neutrophil percentage, median (IQR), %	76.6 (65.9–83.7)	72.4 (62.1–80.2)	82.2 (74.4–88.3)	<0.001
Albumin, mean (SD), g/L	42.3 (4.6)	43.7 (4.1)	39.6 (4.4)	<0.001
NPAR, mean (SD)	1.8 (0.4)	1.6 (0.4)	2.1 (0.4)	<0.001
CRP, median (IQR), mg/L	2.2 (0.6–7.8)	1.4 (0.5–4.4)	4.5 (1.5–16.1)	<0.001
Serum anti-NMDAR antibody, n (%)	75/116 (64.7)	46/71 (64.8)	29/45 (64.4)	0.97
**Clinical features and treatment from onset to discharge**				
Time from onset to admission, median (IQR)	15 (8–26)	15 (7.3–30)	15 (8–23)	0.629
Anti-infective therapy before admission, n (%)	85 (47)	50 (41.7)	35 (57.4)	0.045
Prodromal symptoms, n (%)	114 (63)	75 (62.5)	39 (63.9)	0.85
mRS upon admission, median (IQR)	3 (2–4)	3 (2–4)	4 (4–5)	<0.001
Seizures, n (%)	105 (58)	62 (51.7)	43 (70.5)	0.015
Psychiatric behavior, n (%)	148 (81.8)	96 (80.0)	52 (85.2)	0.388
Involuntary movement, n (%)	28 (15.5)	15 (12.5)	13 (21.3)	0.121
Autonomic dysfunction, n (%)	117 (64.6)	70 (58.3)	47 (77)	0.013
Consciousness declination, n(%)	106 (58.6)	49 (40.8)	57 (93.4)	<0.001
Cognitive dysfunction, n (%)	69 (38.1)	47(39.2)	22 (36.1)	0.685
Speech dysfunction, n (%)	32 (17.7)	21 (17.5)	11 (18)	0.929
ICU admission, n (%)	107 (59.1)	49 (40.8)	58 (95.1)	<0.001
Tumor presence, n (%)	15 (8.3)	8 (6.7)	7 (11.5)	0.267
Pneumonia, n (%)	76 (42)	36 (30)	40 (65.6)	<0.001
First line immunotherapy, n (%)	173 (95.6)	115 (95.8)	58 (95.1)	0.816
Two or more first-line immunotherapies, n (%)	119 (65.7)	74 (61.7)	45 (73.8)	0.105
Second line immunotherapy, n (%)	13 (7.2)	10 (8.3)	3 (4.9)	0.4
Hospital stay, median (IQR)	27 (18–40.5)	26.5 (18–39)	31 (17.5–43)	0.766

mRS, modified Rankin scale; IQR, interquartile range; MRI, magnetic resonance imaging; CSF, cerebrospinal fluid; WBC, count of white blood cell; SD, standard deviation; NPAR, neutrophil percentage-to-albumin ratio; CRP, C-reactive protein; NMDAR, N-methyl-D-aspartate receptor; ICU, intense care unit.

### Comparison of Clinical Data in Anti-MNDAR Encephalitis With Different Disease Severity at Admission and Short-Term Prognosis

**Figure 2:**
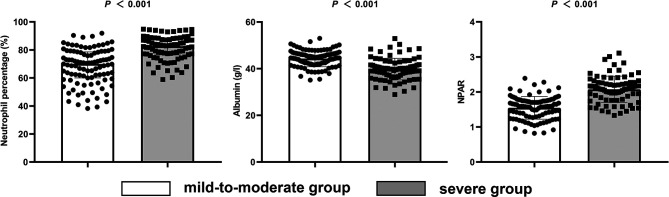
Levels of neutrophil percentage, albumin, and NPAR in the mild-to-moderate and severe admission groups.

Patients were divided into a mild-to-moderate group (mRS ≤ 3) and a severe group (mRS > 3) according to their mRS at admission. The results showed that autonomic dysfunction, consciousness declination, ICU admission, WBC, CRP, neutrophil percentage, albumin, and NPAR were statistically significantly different between the two groups (*P* < 0.05) (**Table 1**). More patients in the severe group had high NPAR compared to the mild-to-moderate group, suggesting that high NPAR may be related to the more severe condition at admission (**Figure 2**). Gender, age, time from onset to admission, anti-infective therapy before admission, prodromal symptoms, seizures, psychiatric behavior, involuntary movement, cognitive dysfunction, speech dysfunction, tumor presence, abnormal MRI, CSF tests, and serum anti-NMDAR antibody were not statistically different between the two groups. (*P* > 0.05) (**Table 1**).

**Figure 3:**
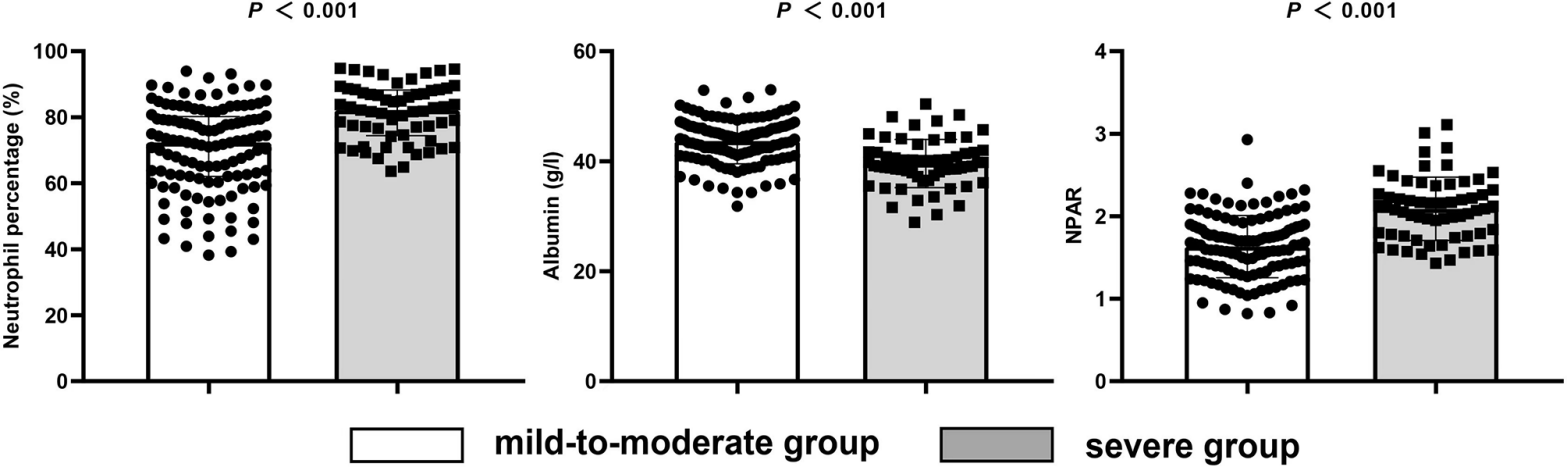
Levels of neutrophil percentage, albumin, and NPAR in the mild-to-moderate and severe short-term prognosis groups.

The patients were divided into a mild-to-moderate group (mRS ≤3) and a severe group (mRS >3) according to their mRS at discharge. Demographic data, auxiliary examination, and all the clinical features from onset to charge were included in the analysis. The results showed that there were statistically significant differences between the two groups in terms of WBC, neutrophil percentage, albumin, NPAR, CRP, anti-infective therapy before admission, mRS score upon admission, seizures, autonomic dysfunction, consciousness declination, ICU admission, and pneumonia (*P* < 0.05). More patients in the severe group had high NPAR compared to the mild-to-moderate group, suggesting that high NPAR may be related to the poor short-term prognosis in patients with anti-NMDAR encephalitis (**Figure 3**). There was no statistically significant difference between the two groups in terms of gender, age, abnormal MRI, CSF tests, time from onset to admission, prodromal symptoms, psychiatric behavior, involuntary movement, cognitive dysfunction, speech dysfunction, tumor presence, immunotherapy, and serum anti-NMDAR antibody (*P* > 0.05) (**Table 2**).

### Multivariable Logistic Regression of Disease Severity at Admission and Short-Term Prognosis

**Figure 4:**
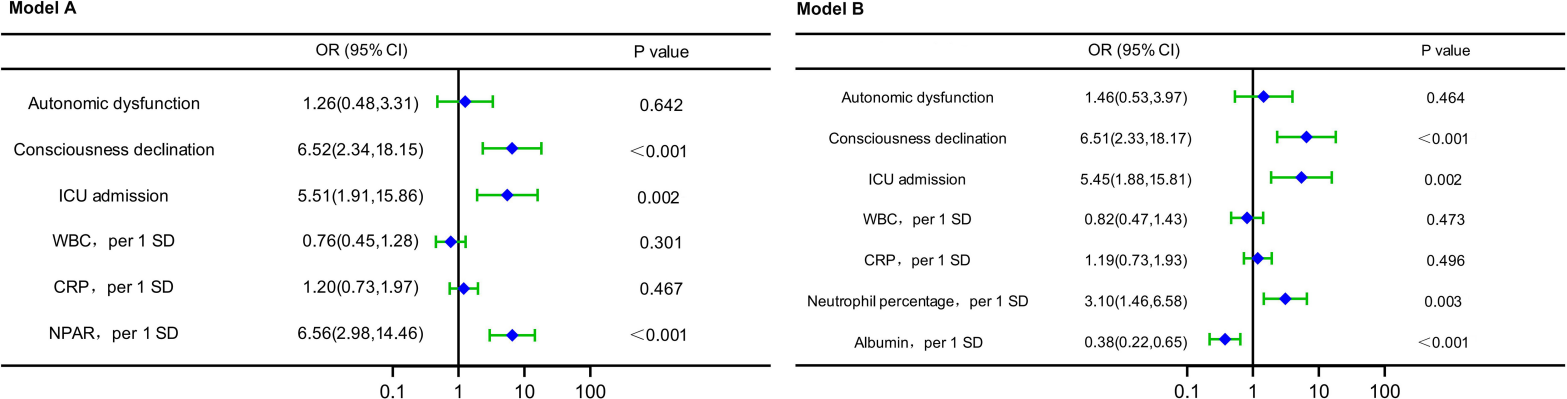
Forest plots of multivariable logistic regression affecting disease severity at admission (Model A and Model B). OR, odds ratio; CI, confidence interval; ICU, intense care unit; WBC, count of white blood cell; SD, standard deviation; CRP, C-reactive protein; NPAR, neutrophil percentage-to-albumin ratio.

Factors with *P* < 0.05 in **Table 1** were included in the multivariable logistic regression. The variance inflation factor (VIF) of neutrophil percentage, albumin, and NPAR were all >10, and there was serious multicollinearity. Since the NPAR contains two variables, neutrophil percentage and albumin, they were put into the same model, at the same time inevitably leading to multicollinearity. Therefore, it was necessary to build models respectively for multivariable logistic regression, with model A (NPAR) and model B (neutrophil percentage, albumin). Since NPAR is the ratio of neutrophil percentage and albumin, model A and model B are therefore essentially the same, and indeed the multivariable logistic regression of the two models presented the same results. The results showed that ICU admission, consciousness declination, neutrophil percentage, albumin, and NPAR were independent indicators of the disease severity at admission in patients with anti-NMDAR encephalitis (**Figure 4**).

**Figure 5:**
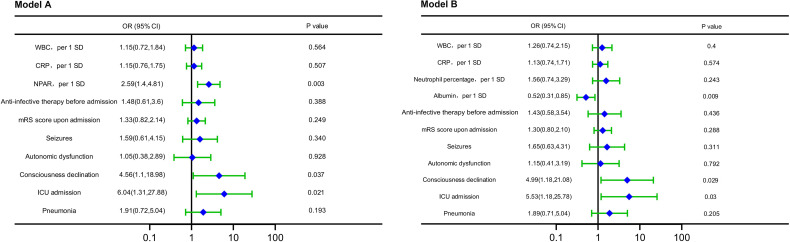
Forest plots of multivariable logistic regression affecting short-term outcome (Model A and Model B). OR, odds ratio; CI, confidence interval; WBC, count of white blood cell; SD, standard deviation; CRP, C-reactive protein; NPAR, neutrophil percentage-to-albumin ratio; mRS, modified Rankin scale; ICU, intense care unit.

Factors with *P* < 0.05 in **Table 2** were included in the multivariable logistic regression. The VIF of neutrophil percentage, albumin, and NPAR were all >10, and there was serious multicollinearity. Since the NPAR contains two variables, neutrophil percentage and albumin, they were put into the same model, at the same time inevitably leading to multicollinearity. Therefore, it was necessary to build models respectively for multivariable logistic regression, with model A (NPAR) and model B (neutrophil percentage, albumin). Since NPAR is the ratio of neutrophil percentage and albumin, model A and model B are therefore essentially the same, and indeed the multivariable logistic regression of the two models presented the same results. The results showed that ICU admission, consciousness declination, albumin, and NPAR were independent indicators of short-term prediction in patients with anti-NMDAR encephalitis (**Figure 5**).

### ROC Curves of Individual Factors for Assessing Disease Severity at Admission and Predicting Short-Term Prognosis

**Figure 6:**
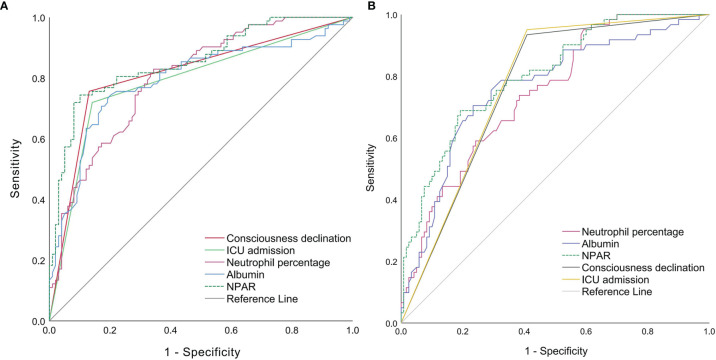
ROC curves of individual factors in assessing severity at admission **(A)** and predicting short-term prognosis **(B)** ICU, intense care unit; NPAR, neutrophil percentage-to-albumin ratio.

The variables with *P* < 0.05 in the multivariable logistic regression analysis were included in the ROC curves (**Figure 6A** and **Table 3**), and in the assessment of factors influencing disease severity at admission, the AUC of NPAR was significantly higher than that of neutrophil percentage and albumin (*P*=0.01, *P*=0.019), but comparable to those of consciousness declination and ICU admission (*P*=0.295, *P*=0.106). With a cut-off value of 1.88, NPAR showed a sensitivity of 74%, and a specificity of 90%.

**Table 3:** Comparison of AUC values between NPAR and other factors in assessing severity at admission.

	AUC	95%CI	*P* value
Consciousness declination	0.812	0.746–0.879	0.295
ICU admission	0.789	0.719–0.859	0.106
Neutrophil percentage	0.8	0.737–0.863	0.01
Albumin	0.794	0.725–0.863	0.019
NPAR	0.854	0.798–0.91	

AUC, area the under curve; CI, confidence interval; ICU, intense care unit; NPAR, neutrophil percentage-to-albumin ratio.

The variables with *P* < 0.05 in the multivariable logistic regression analysis and neutrophil percentage were included in the ROC curves (**Figure 6B** and **Table 4**), and in the factors predicting short-term outcome, the AUC of NPAR was significantly higher than that of neutrophil percentage (*P*=0.009), but comparable to those of albumin (*P*=0.128), consciousness declination (*P*=0.354) and ICU admission (*P*=0.43). With a cut-off value of 1.95, NPAR showed a sensitivity of 69%, and a specificity of 81%.

**Table 4:** Comparison of AUC values between NPAR and other factors in predicting short-term outcome.

	AUC	95% CI	*P* value
Neutrophil percentage	0.745	0.673–0.817	0.009
Albumin	0.762	0.687–0.837	0.128
NPAR	0.803	0.737–0.868	
Consciousness declination	0.763	0.694–0.833	0.354
ICU admission	0.771	0.703–0.839	0.430

AUC, area the under curve; CI, confidence interval; ICU, intense care unit; NPAR, neutrophil percentage-to-albumin ratio.

**Figure 7:**
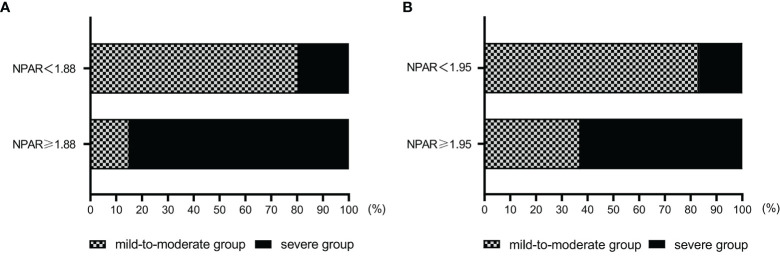
**(A)** Distribution of patients with different severity at admission grouped by the NPAR cutoff value. **(B)** Distribution of patients with different short-term prognosis grouped by the NPAR cutoff value. NPAR, neutrophil percentage-to-albumin ratio.

Multivariable logistic regression analysis showed that the OR of NPAR was >1, suggesting that NPAR was a factor contributing to exacerbation. We divided the patients into 2 groups using a cutoff value of 1.88 for NPAR as the node and analyzed the distribution of patients with different severity at admission in the two groups. The results showed that the proportion of patients in the mild-to-moderate group was higher in the NPAR <1.88 group and the proportion of patients in the severe group was higher in the NPAR ≥1.88 group (**Figure 7A**).

Multivariable logistic regression analysis showed that the OR of NPAR was >1, suggesting that NPAR was a factor contributing to exacerbation. We divided the patients into 2 groups using a cutoff value of 1.95 for NPAR as the node and analyzed the distribution of patients with different short-term outcomes in the two groups. The results showed that the proportion of patients in the mild-to-moderate group was higher in the NPAR <1.95 group and the proportion of patients in the severe group was higher in the NPAR ≥1.95 group (**Figure 7B**).

### ROC Curves of the Multivariable Logistic Regression Models

**Figure 8:**
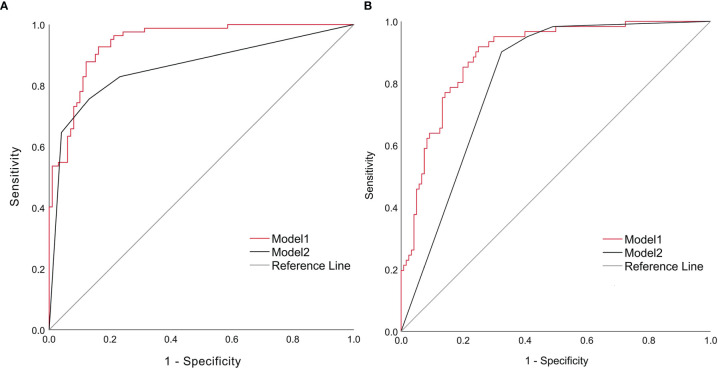
ROC curves for assessing disease severity at admission **(A)** and predicting short-term prognosis **(B)** ROC, receiver operating characteristic.

The ROC curves of the models for assessing disease severity at admission constructed from multiple independent influences were shown in **Figure 8A**, with an AUC of 0.940 (95% CI: 0.902, 0.966) for model 1 (with NPAR) and 0.859 (95% CI: 0.806,0.937) for model 2 (without NPAR). The model with NPAR showed superior sensitivity and specificity, and was a better assessment model.

The ROC curves of the models for predicting short-term outcome constructed from multiple independent influences were shown in **Figure 8B**, with smoothed AUC of 0.885 (95% CI: 0.831, 0.934) for model 1 (with NPAR) and 0.836 (95% CI: 0.718, 0.940) for model 2 (without NPAR). The model with NPAR showed superior sensitivity and specificity and was a better prediction model.

### Nomograms and Bootstrap Validation of the Multivariable Logistic Regression Models

**Figure 9:**
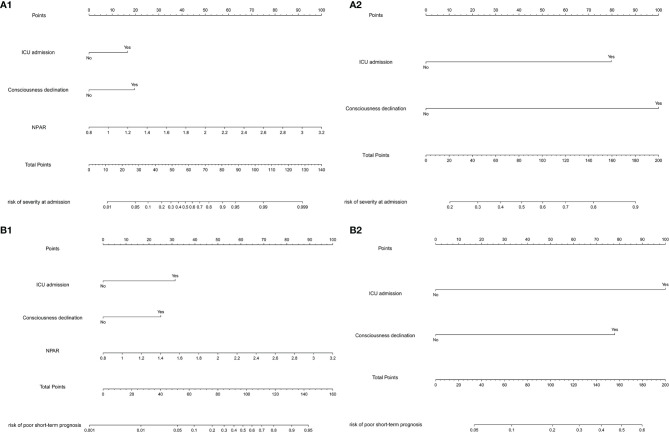
**(A1)** Nomogram including NPAR for assessing disease severity at admission. **(A2)** Nomogram without NPAR for assessing severity at admission. **(B1)** Nomogram including NPAR for predicting short-term prognosis. **(B2)** Nomogram without NPAR for predicting short-term prognosis.

According to the nomograms of the multivariable logistic regression models to assess disease severity at admission (**Figures 9A1, A2**) and validated by bootstrap, the c-index was 0.940 (95% CI: 0.887–0.996) when NPAR was included and decreased to 0.859 (95% CI: 0.751–0.996) when NPAR was removed. The multivariable logistic regression model including NPAR had good consistency with the actual situation and had better assessment efficacy.

According to the nomograms of the multivariable logistic regression models to predict short-term prognosis (**Figures 9B1, B2**) and validated by bootstrap, the c-index was 0.892 (95% CI: 0.797–0.986) when NPAR was included and decreased to 0.808 (95% CI: 0.706–0.909) when NPAR was removed. The multivariable logistic regression model including NPAR had good consistency with the actual situation and had better assessment efficacy.

### Calibration Curves of Multivariable Logistic Regression Models

**Figure 10:**
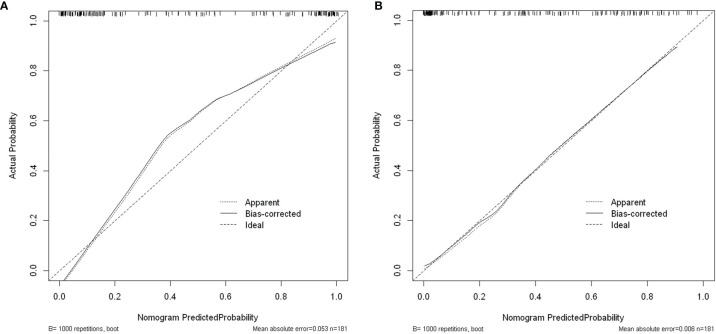
**(A)** Calibration curves for assessing disease severity at admission. **(B)** Calibration curves for predicting short-term prognosis.

Bootstrap was performed for 1000 cycles to validate the nomogram models constructed with multiple independent influences such as NPAR. Calibration curves were used to compare the assessment of disease severity (**Figure 10A**) and short-term outcome (**Figure 10B**) between the nomograms and actual observation. The calibration curves revealed good predictive accuracy of the nomograms. The Hosmer–Lemeshow goodness-of-fit tests showed that the models had good consistency in the assessment of disease severity at admission (χ^2^ = 12.725, *P* = 0.122) and short-term outcome (χ^2^ = 6.012, *P* = 0.646).

### Decision Curve Analysis of Multivariable Logistic Regression Models

**Figure 11:**
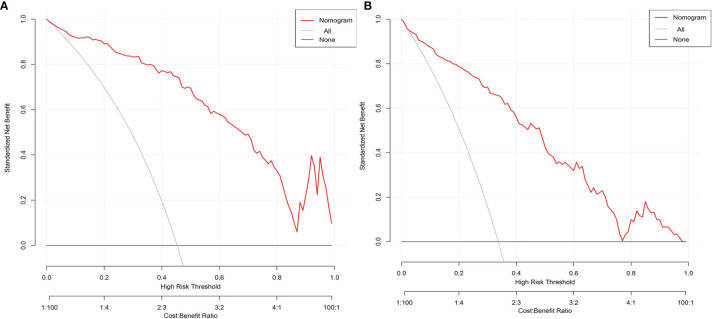
**(A)** Decision curve analysis for assessing disease severity at admission. **(B)** Decision curve analysis for predicting short-term prognosis.

Multiple independent influencing factors such as NPAR were constructed as models, with the applicability and validity evaluated by decision curve analysis. Results showed that the constructed multivariable logistic regression models for assessing disease severity at admission (**Figure 11A**) and predicting short-term prognosis (**Figure 11B**) had high net benefit values, showing good applicability and validity.

## Discussion

Anti-NMDAR encephalitis is the most common autoimmune encephalitis, which can lead to disability and death in severe cases. Early evaluation of the condition and prognosis and reasonable treatment will help to reduce disability and improve the prognosis. However, it still lacks biological indicators that can better evaluate the severity and predict the prognosis of anti-NMDAR encephalitis. Therefore, the current study was performed to explore the potential biological indicators that were predictive of the severity at admission and short-term prognosis.

In this study, the clinical data of 181 patients in our hospital were retrospectively analyzed, and it was found that two groups of patients with anti-MNDAR encephalitis of different severity at admission had significant statistical differences in autonomic dysfunction, consciousness declination, ICU admission, WBC, CRP, neutrophil percentage, albumin, and NPAR. More patients in the severe group had high NPAR compared to the mild-to-moderate group, suggesting a correlation between NPAR and disease severity. Two groups of anti-MNDAR encephalitis patients with different short-term outcomes had significant statistical differences in anti-infective therapy before admission, mRS score upon admission, seizures, autonomic dysfunction, consciousness declination, pneumonia, ICU admission, WBC, CRP, neutrophil percentage, albumin, and NPAR. More patients in the severe group had high NPAR compared to the mild-to-moderate group, suggesting a correlation between NPAR and short-term prognosis. 

ROC curves were drawn for comparative analysis of related indicators and demonstrated that the AUC of NPAR was the largest among multiple independent influencing factors, suggesting that NPAR, a good indicator for assessing the severity at admission and predicting short-term prognosis, had excellent sensitivity and specificity. OR of NPAR was greater than 1, suggesting that NPAR was a factor for promoting the exacerbation of the disease. The cut-off values of NPAR for assessing the severity at admission and predicting short-term prognosis were 1.88 and 1.95 respectively. The grouping based on the cutoff value showed that the proportion of mild-to-moderate patients in the group with NPAR <1.88 was higher, and the proportion of severe patients in the group with NPAR ≥1.88 was higher, suggesting that a higher NPAR indicated more serious situation at admission. The grouping based on the cutoff value showed that the proportion of mild-to-moderate patients in the group with NPAR <1.95 was higher, and the proportion of severe patients in the group with NPAR ≥1.95 was higher, suggesting that the higher NPAR indicated poor short-term prognosis. Among the assessment models constructed from multiple influencing factors, the AUC of the ROC curves of the model including NPAR was better and had a better discrimination. Nomograms of the model based on risk factors such as NPAR had better agreement among nomograms of assessing disease severity at admission and predicting short-term prognosis with the actual situation, and the C-index of the nomograms decreased when NPAR was deleted, so the assessment efficacy decreased. The calibration curves of the multivariable logistic regression analysis models constructed by NPAR and others were in agreement with the standard curves, and the results of the Hosmer–Lemeshow goodness-of-fit tests showed that the models had good calibration ability. The decision curve analysis showed that the multivariable logistic regression analysis models constructed by NPAR and others had a high net benefit value, which displayed good applicability and validity.

The neutrophil, as an important component of leukocytes and a key effector cell, plays an important role in mediating the inflammatory response. At the same time, the neutrophil, an important component of the innate immune system, is the first subset of immunoreactive cells to be attacked by antigens. Neutrophils can also promote the activation of other immune cells and induce cellular immunity and humoral immunity (8, 9). It can be seen that neutrophils mediate the inflammatory response; and they also participate in and affect multiple links of the immune response. Some studies have also confirmed that neutrophils play an important role in immune diseases such as Guillain–Barre syndrome (10), multiple sclerosis (11), and systemic lupus erythematosus (12).

Serum albumin is a kind of multifunctional non-glycosylated plasma protein. The strong binding capacity of albumin can reduce the free concentration of toxic metabolites and reduce the pathophysiological damage caused by the poison (13). Albumin can downregulate the expression and transport of inflammatory factors and reduce the inflammatory cascade reaction (14). Albumin can also affect the pharmacokinetics of many drugs and thus the efficacy of drugs (15). Some studies have also confirmed that albumin is a factor affecting the progression of immune diseases such as Guillain–Barre syndrome (16) and autoimmune encephalitis (2).

It can be seen that the physiological functions of neutrophils and albumin make them play pivotal roles in immune diseases. Albumin stimulates the voltage-gated proton channel in neutrophils and affects the generation of reactive oxygen species (ROS) and the release of elastase (17). ROS can change the protein structure of host organs and tissues, expose new epitopes, and affect immune tolerance (18). It can be seen that there is also an interaction between albumin and neutrophils. NPAR combines both neutrophils and albumin and integrates different factors of inflammation and immune response. Compared with a single factor, NPAR better reflects the state of the systemic immune response and inflammatory response. The changes of NPAR can better reflect the damaged degree of immune inflammation homeostasis and disease activity, and it is an ideal biological index. However, to date, no study has reported the association of NPAR with anti-NMDAR encephalitis. At our center, 181 cases of anti-NMDAR encephalitis were studied. NPAR was first confirmed to have good clinical value in evaluating the severity of anti-NMDAR encephalitis at admission and predicting short-term outcome.

The incidence of anti-NMDAR encephalitis is low. Most of the related published articles are small-sample studies of dozens of cases. In this study, 181 anti-NMDAR encephalitis patients were finally included, and the sample size was relatively large. This is mainly because our region is the most populous province in China (at 00:00 on November 1, 2020, the results of the 7th national population census: the permanent population of Henan Province was 99,365,519). Our hospital is located in the provincial capital city of the center of our region, and it is the largest hospital with the strongest comprehensive medical level in the province, so that our patients are from all over the province. However, the single-center study has limited this study. In addition, the retrospective study also makes the data collection with certain limitations. For example, the ROC curve showed that the NPAR at admission had a very high sensitivity and specificity for the assessment of the severity of the admission condition. Although NPAR at the time of admission also had a good sensitivity and specificity for the prediction of the short-term outcome, it was slightly decreased compared with its assessment of the severity at admission. The nomogram also showed that the NPAR at the time of admission had a very good evaluation efficiency for the severity of disease at admission. Although the NPAR at admission had a good prediction efficiency for the disease severity at discharge, its efficiency was slightly decreased compared with its evaluation for the severity of the admitted condition. There might be a closer correlation between NPAR at discharge and mRS at discharge. However, due to the limitations of retrospective studies, some patients were not reexamined for neutrophils and albumin at discharge and statistical analysis could not be performed. In fact, our patients’ neutrophils and albumin as well as the severity of disease were dynamically changing, and the results would be more convincing if we could observe the dynamic correlations between NPAR and mRS. If prospective multicenter and large-sample research is conducted to dynamically observe the changes of NPAR and mRS, as well as their correlation, perhaps more convinced and exact conclusions can be drawn.

In conclusion, although there were some limitations in this study, it was a beneficial exploration and attempt in the clinical study of anti-NMDAR encephalitis. We found that NPAR has an association with the severity at admission and short-term outcome in patients with anti-NMDAR encephalitis. This might provide reference and enlightenment for the further study. In addition, the accessibility of NPAR detection and calculation is very good, which facilitates the potential broad use of NPAR in the future.

## Data Availability Statement

The raw data supporting the conclusions of this article will be made available by the authors, without undue reservation.

## Ethics Statement

The studies involving human participants were reviewed and approved by the Ethics Committee of the First Affiliated Hospital of Zhengzhou University (number: 2021-KY-0974-002). Written informed consent from the participants’ legal guardian/next of kin was not required to participate in this study in accordance with the national legislation and the institutional requirements.

## Author Contributions

YT wrote the main manuscript, analyzed the data, and prepared the tables and figures. HH, LL, LiuY, SZ, LuY, XH, and JW collected the data. JW designed the research, led the research group, and arranged the work of all authors. All authors reviewed the manuscript. All authors contributed to the article and approved the submitted version.

## Funding

This work was supported by the National Natural Science Foundation of China to JW (grant number U1604181), the Joint project of Medical science and Technology Research Program of Henan Province to JW (grant number LHGJ20190078), Henan Medical Education Research Project to JW (grant number Wjlx2020531), and Henan Province Key R&D and Promotion Special Project (Science and Technology Tackle) to JW (grant number 212102310834).

## Conflict of Interest

All authors declare that the research was conducted in the absence of any commercial or financial relationships that could be construed as a potential conflict of interest.

## Publisher’s Note

All claims expressed in this article are solely those of the authors and do not necessarily represent those of their affiliated organizations, or those of the publisher, the editors and the reviewers. Any product that may be evaluated in this article, or claim that may be made by its manufacturer, is not guaranteed or endorsed by the publisher.
